# Editorial: The Role of Stem Cells, Epigenetics and MicroRNAs in Parkinson's Disease

**DOI:** 10.3389/fnins.2020.00515

**Published:** 2020-06-16

**Authors:** Shane V. Hegarty, Holly F. Green, Jonathan Niclis, Gerard W. O'Keeffe, Aideen M. Sullivan

**Affiliations:** ^1^Department of Neurology, F.M. Kirby Neurobiology Center, Boston Children's Hospital, Harvard Medical School, Boston, MA, United States; ^2^Department of Basic and Clinical Neuroscience, Institute of Psychiatry, Psychology and Neuroscience, King's College London, London, United Kingdom; ^3^Novo Nordisk, Copenhagen, Denmark; ^4^Department of Anatomy and Neuroscience, University College Cork (UCC), Cork, Ireland; ^5^Cork Neuroscience Centre, University College Cork, Cork, Ireland

**Keywords:** Parkinson's disease, stem cells, epigenetics, microRNA, therapy

Parkinson's disease (PD) is a common, age-related and progressive neurodegenerative disorder that affects 1–2% of people over 65. As mean population ages and life expectancies increase globally, the incidence of PD is projected to double by 2030. Two classical hallmarks of PD are the degeneration of substantia nigra (SN) dopaminergic (DA) neurons, and the presence of Lewy bodies in CNS and PNS subregions. Despite decades of research our understanding of the pathophysiology and the diagnosis of PD remains limited, while no disease-modifying therapies presently exist. Current dopamine-replacement strategies and surgical interventions can provide symptomatic relief. However, these symptomatic treatments do not arrest or reverse the underlying pathology, their effectiveness wanes with time, and they typically produce disabling side effects (Poewe et al., 2017). As such, there is an urgent clinical need to better understand and diagnose PD, and to develop novel disease-modifying therapies for PD.

A promising disease-modifying therapy involves the replacement of lost SN DA neurons with stem cell-derived replicas (Stoker et al.; Parmar et al., 2020). Such neurons, particularly when derived from PD patients, can also be used to model and investigate the human pathology, and/or to evaluate novel therapies/drugs in PD. Furthermore, there is increasing evidence that epigenetic disturbances and dysregulated microRNA expression occur in PD. Such changes may contribute to the underlying pathophysiology, and could serve as PD biomarkers or even provide novel pharmacological targets (Hegarty et al., 2016; Singh and Sen, 2017; Stoker et al.; van Heesbeen and Smidt). This special issue aimed to publish high-quality articles covering the use of stem cells, epigenetics, and/or microRNA to better understand and diagnose PD, and to ultimately advance research with the potential to produce novel treatments for PD. In this regard, we have gathered six articles which describe the emergence of circulating microRNAs as PD biomarkers, using epigenetics to understand and model PD pathogenesis, and the promise of stem cell-based therapies to treat PD patients.

Chronologically, the first article “*Circulating miRNAs as Diagnostic Biomarkers for Parkinson's Disease*” by Roser et al. discusses the applicability of different body fluids as sources for microRNA biomarkers, highlights technical considerations for microRNA analyses, and then gives an overview of the current evidence on circulating microRNAs as biomarker candidates for PD patients. This mini-review provides a rich resource of information for those aiming to mine the human miRNAome to uncover novel, accessible and reliable diagnostics, or even prognostics, for PD. Indeed, in 2019 Yang et al. published their research article on “*Elevated Plasma microRNA-105-5p Level in Patients With Idiopathic Parkinson's Disease: A Potential Disease Biomarker*.” In this study, the authors used a combined bioinformatics and biomarker discovery approach to identify and characterize a novel miRNA biomarker of PD. Specifically they used existing miRNA (GSE16658) (Martins et al., 2011) and mRNA expression data (GSE6613) (Scherzer et al., 2007) from PD patients to construct a PD-specific microRNA-mRNA expression network of differentially expressed microRNA-RNA. This approach revealed that miR-105-5p may be a putative biomarker for PD. Yang et al. then experimentally validated that miR-105-5p is elevated in the plasma of idiopathic PD patients, compared to healthy controls and non-PD neurological disorder controls. Moreover, they demonstrated that the miR-105-5p microRNA biomarker was unaffected by L-DOPA, and dopamine receptor agonists, treatments in idiopathic PD patients. Thus, in a clinical setting this microRNA biomarker seems unaffected by a patient's medication status, and can distinguish idiopathic PD from other neurological disorders. Taken together, these two articles demonstrate the utility and promise of circulating microRNAs as PD biomarkers.

Next in 2019, van Heesbeen and Smidt published the review article “*Entanglement of Genetics and Epigenetics in Parkinson's Disease*,” in which the authors point out important difficulties in untangling the genetic and epigenetic mechanisms that may underlie PD progression. Despite this, they describe a number of solid examples which demonstrate independent roles for epigenetic mechanisms, such as age-related epigenetic drift, in PD. In particular, van Heesbeen and Smidt review multiple lines of evidence showing epigenetic (dys)regulation of the *SNCA* gene, one of the hallmark PD genes. Finally, the authors discuss several mechanisms that may affect local and global epigenetics in PD neurons, including inflammation, oxidative stress, autophagy, and DNA repair mechanisms, and hypothesize how characterizing the PD epigenome may lead to future therapeutic targets. Complimentary to this review, a mini-review by De Boni and Wüllner entitled “*Epigenetic Analysis in Human Neurons: Considerations for Disease Modeling in PD*” explores how using humanized disease models, such as induced pluripotent stem cells (iPSC)-derived DA neurons from PD patients, can facilitate the study of epigenetic alterations in PD. They highlight the current advantages and limitations of these models, such as whether the epigenetic (age-associated) landscape of human *in vitro* neurons compare to the affected neurons in PD patients. In this review, In this review, De Boni and Wüllner demonstrate how human neuronal PD model systems are being used to characterize epigenetic modifications in PD, for example by correlating epigenetic changes to gene expression alterations, and how these insights may inform the development of novel PD therapeutics in the future. Taken together, these two articles comprehensively review the recent progresses toward understanding the contribution of epigenetics to PD pathogenesis.

In 2020, the original research article by Precious et al. entitled “*Dopaminergic Progenitors Derived From Epiblast Stem Cells Function Similarly to Primary VM-Derived Progenitors When Transplanted Into a Parkinson's Disease Model*” compared stem cell sources for DA transplants. To do this they employed an allograft system where the DA progenitor cells derived from two cell sources, mouse epiblast stem cells, and primary fetal mouse ventral mesencephalon (VM) tissue, were transplanted into the striatum of 6-OHDA lesioned mice pre-treated with L-DOPA, a widely-used PD rodent model. Precious et al. then assessed standard behavioral tests for functional improvements, such as drug-induced rotations, a number of motor tests and drug-induced abnormal involuntary movements. Following these behavioral analyses, the authors reported no significant differences in the functional restoration ability between the gold standard primary VM-derived DA transplants, compared to the pluripotent stem cell-derived DA transplants. This is an important finding for the stem cell neurotransplantation field in PD, as pluripotent stem cell sources have many benefits over primary fetal tissue, such as supply, scalability, consistency, and suitability for good manufacturing practices. Finally, the promise of stem cell-based therapies for PD was comprehensively reviewed in the article “*Emerging Treatment Approaches for Parkinson's Disease*” by Stoker et al.. In addition to discussing these regenerative cell-based therapies in great detail, this review also explores how gene therapies are being designed to treat the DA aspects of PD, whilst limiting adverse effects, as well as novel approaches to reduce α-synuclein pathology. This mini-review offers an optimistic perspective on the future of PD therapy, by describing how a number of exciting treatments have yielded promising results in pre-clinical and early clinical trials. Indeed, Stoker et al. provided an appropriate conclusion to this special issue when they wrote “it now seems likely that the landscape for the management of PD will change dramatically in the short to medium term future.”

As a whole, this special issue represents the current state-of-the-art in our understanding of the role of stem cells, epigenetics, and microRNAs in PD. We would like to thank all the authors for their valuable participation in this Research Topic, and we hope that it becomes a major reference point and resource for those working in the PD field.

## Author Contributions

SH, GO'K, and AS wrote the article, which was then reviewed by all authors.

## Conflict of Interest

JN was employed by company Novo Nordisk. The remaining authors declare that the research was conducted in the absence of any commercial or financial relationships that could be construed as a potential conflict of interest.
